# Authentication of the Herbal Medicine Angelicae Dahuricae Radix Using an ITS Sequence-Based Multiplex SCAR Assay

**DOI:** 10.3390/molecules23092134

**Published:** 2018-08-24

**Authors:** Pureum Noh, Wook Jin Kim, Sungyu Yang, Inkyu Park, Byeong Cheol Moon

**Affiliations:** Herbal Medicine Research Division, Korea Institute of Oriental Medicine, Daejeon 34054, Korea; pureum322@kiom.re.kr (P.N.); ukgene@kiom.re.kr (W.J.K.); sgyang81@kiom.re.kr (S.Y.); pik6885@kiom.re.kr (I.P.)

**Keywords:** *Angelica dahurica*, *Angelica dahurica* var. *formosana*, internal transcribed spacer (ITS), sequence-characterized amplified region (SCAR) marker, molecular authentication, multiplex polymerase chain reaction (multiplex PCR)

## Abstract

The accurate identification of plant species is of great concern for the quality control of herbal medicines. The Korean Pharmacopoeia and the Pharmacopoeia of the People’s Republic of China define Angelicae Dahuricae Radix (*Baek-Ji* in Korean and *Bai-zhi* in Chinese) as the dried roots of *Angelica dahurica* or *A. dahurica* var. *formosana* belonging to the family Apiaceae. Discrimination among *Angelica* species on the basis of morphological characteristics is difficult due to their extremely polymorphic traits and controversial taxonomic history. Furthermore, dried roots processed for medicinal applications are indistinguishable using conventional methods. DNA barcoding is a useful and reliable method for the identification of species. In this study, we sequenced the internal transcribed spacer (ITS) region of nuclear ribosomal RNA genes in *A. dahurica*, *A. dahurica* var. *formosana*, and the related species *A. anomala* and *A. japonica*. Using these sequences, we designed species-specific primers, and developed and optimized a multiplex sequence-characterized amplified region (SCAR) assay that can simply and rapidly identify respective species, and verify the contamination of adulterant depending on the polymerase chain reaction (PCR) amplification without sequencing analysis in a single PCR reaction. This assay successfully identified commercial samples of Angelicae Dahuricae Radix collected from Korean and Chinese herbal markets, and distinguished them from adulterants. This multiplex SCAR assay shows a great potential in reducing the time and cost involved in the identification of genuine Angelicae Dahuricae Radix and adulterant contamination.

## 1. Introduction

Herbal medicines have been used for thousands of years to promote human health and cure diseases. Nowadays, herbal medicines occupy an indispensable position in the medical treatment of human diseases across the world. The market for herbal medicinal products has increased substantially and consistently in past years [1]. Because the efficacy and safety of herbal medicines is highly dependent on the proper use of authentic material, the accurate identification of herbs is a major concern [2]. However, discriminating between species used for herbal medicines and related species is very difficult, due to similarities in the morphology of processed tissues, such as roots, stems, leaves, fruits, and seeds [3,4].

In the past, the authentication of the ingredients of herbal medicines has relied on personal skills and has lacked objectivity and accuracy [4]. However, over the last two decades, technological advances in molecular genetic tools have offered highly efficient and reliable DNA-based methods of herbal authentication. Species-specific differences in sequences of short DNA fragments, referred to as DNA barcodes, provide researchers with a rapid, effective, and accurate method for species identification [5,6]. DNA barcoding is used to distinguish between authentic herbal products and adulterants [2,7,8]. The Consortium for the Barcode of Life (CBOL), an international initiative working group which aims to develop a standard region of DNA barcoding as a tool for species identification, has developed and suggested diverse DNA barcode regions for identifying plant species [9]. To date, several candidate regions have been used for the identification of plant species, including maturase K (*matK*), ribulose-1,5-bisphospate carboxylase/oxygenase large subunit (*rbcL*), *psbA*-*trnH* intergenic spacer region (*psbA*–*trnH*), internal transcribed spacer (ITS), *trnL-trnF* intergenic spacer region (*trnL-F*), and 5S and 18S ribosomal RNAs (rRNAs) [2]. Among these, two chloroplast genes, *matK* and *rbcL*, and the ITS region of nuclear ribosomal DNA (rDNA), have been commonly used as DNA barcodes in plants [10,11,12,13].

According to the Korean Pharmacopoeia and the Pharmacopoeia of the People’s Republic of China, Angelicae Dahuricae Radix (*Baek-Ji* in Korean and *Bai-zhi* in Chinese) refers to the dried roots of *Angelica dahurica* (Hoffm.) Benth. & Hook.f. ex Franch. & Sav. or *A. dahurica* var. *formosana* (Boissieu) Yen in the family Apiaceae [14,15,16,17]. This herb is commonly used to treat pain, abscesses, furunculosis, and acne [17]. It is also reported to possess anti-inflammatory, antitumor, antioxidant, and skin whitening properties [18,19,20,21,22,23,24]. However, the authentication of the *Angelica* species for medical purposes is greatly challenging, due to their extremely polymorphic traits and controversial taxonomic history [25]. In particular, *Angelica anomala* Avé-Lall. and *Angelica japonica* A. Gray (Apiaceae) have been frequently misidentified as *A. dahurica* due to their morphological similarity, while the roots of these two species have also been distributed as Angelicae Dahuricae Radix in herbal markets. Therefore, reliable methods to distinguish genuine *A. dahurica* and *A. dahurica* var. *formasana* from other related species are needed, as these could provide significant practical improvements in the quality control of Angelicae Dahuricae Radix.

Sequence-characterized amplified region (SCAR) markers comprise a PCR-based assay to perform DNA barcoding or fingerprinting using sequence-specific primers [3,8]. SCAR markers amplify only the target DNA of interest, and distinguish samples based on the positive or negative amplification of target regions or the length of polymorphisms [8]. SCAR markers permit the identification of herbal medicines in a rapid, simple, cheap, reliable, and reproducible manner [26].

A genetic assay using DNA barcoding has previously been reported for identifying *Angelica* species used as herbal materials in China [25]. However, there are differences in the ranges of plant species that are used as adulterants in herbal medicines, due to differences in the distribution of plant species between countries, and rapid and simple genetic assay methods are required for identifying authentic herbal materials form adulterants. In this study, we designed SCAR markers to simply and rapidly distinguish authentic Angelicae Dahuricae Radix, *A. dahurica*, and *A. dahurica* var. *formosana*, from their common adulterants *A. anomala* and *A. japonica*, on the basis of ITS sequencing. Using these markers, we established a multiplex SCAR assay for the concurrent identification of *Angelica* species, for the monitoring of the distribution of Angelicae Dahuricae Radix in commercial herbal markets.

## 2. Results

### 2.1. Nucleotide Sequence and Phylogenetic Analyses

**Figure 1:**
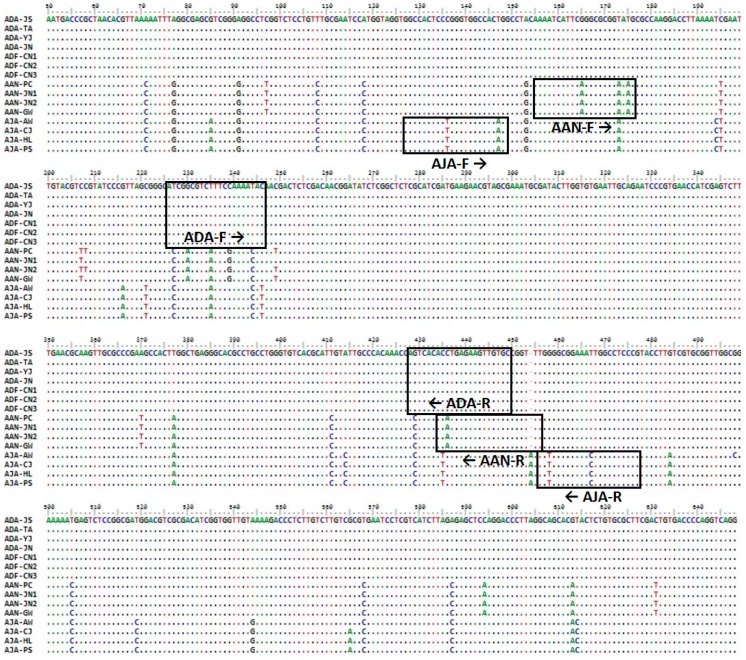
Comparative sequence analysis of the ITS regions in three *Angelica* species. The positions of three species-specific primer pairs used for the development of SCAR markers are outlined in boxes.

Approximately 700 bp of the ITS region was successfully amplified and sequenced from 15 plant samples (Table 1), and the sequence information was deposited in the GenBank database of the National Center for Biotechnology Information (NCBI; see Materials and Methods for accession numbers). The ITS sequences of *A. dahurica* and *A. dahurica* var. *formosana* were 100% identical. The length of the ITS region was 689 bp in *A. dahurica* and *A. anomala* and 690 bp in *A. japonica* (Table 2), and the sequences were aligned to a length of 690 bp. Intra-species variability was zero in *A. dahurica*, 0.0015% ± 0.0012% in *A. anomala*, and 0.0017% ± 0.0012% in *A. japonica* (Table 2). Inter-species variability ranged from 0.0423% ± 0.0021% to 0.0476% ± 0.0053% (Table 2). A species-specific insertion/deletion (indel) mutation was detected at one site in *A. japonica*, and species-specific nucleotide substitutions were detected at 18 sites in *A. dahurica*, 10 sites in *A. anomala*, and 16 sites in *A. japonica* (Table 2). These species-specific nucleotide polymorphisms were used to develop SCAR markers to discriminate among the three *Angelica* species (Figure 1).

We additionally inferred the phylogenetic relationships among the three *Angelica* species related to Angelicae Dahuricae Radix, using ITS sequencing (Figure S1). The Kimura 2-parameter (K2P) model was selected for the phylogenetic analysis. The phylogenetic tree constructed using the maximum likelihood (ML) method revealed a monophyletic group for each species with high bootstrap values (99–100%; Figure S1). Samples of *A. anomala* and *A. japonica* were more closely related with each other than with those of *A. dahurica* (Figure S1). Overall, these data suggest that the three *Angelica* species are identifiable on the basis of sequence variability in the ITS region.

### 2.2. Development of Species-Specific SCAR Markers

**Figure 2:**
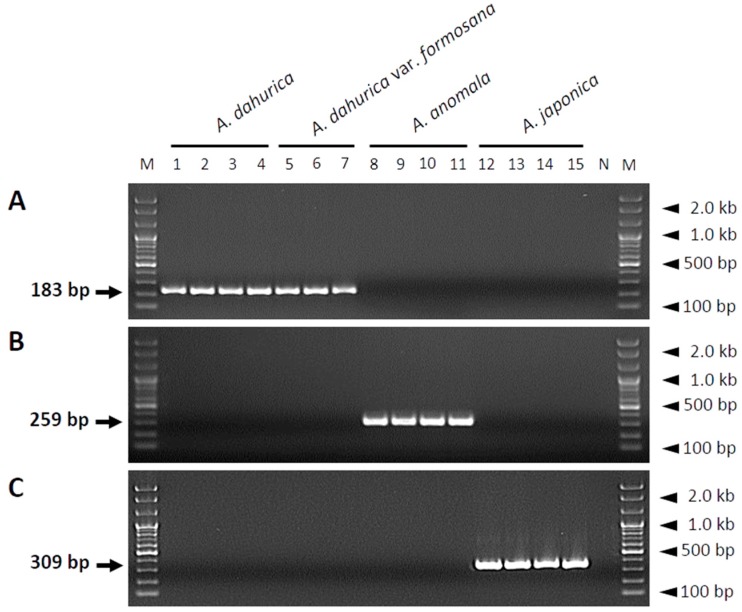
Gel images showing the amplification of SCAR markers developed from sequence variations in the ITS regions of three *Angelica* species using species-specific primer pairs. The ITS sequences of *A. dahurica* (A), *A. anomala* (B), and *A. japonica* (C) were amplified using ADA-F/ADA-R, AAN-F/AAN-R, and AJA-F/AJA-R primer pairs, respectively. Primer sequences are listed in Table 3. Lanes 1, 2, 3, and 4 correspond to samples ADA-JS, ADA-TA, ADA-YJ, and ADA-NJ, respectively, of *A. dahurica*; lanes 5, 6, and 7 correspond to samples ADF-CN1, ADF-CN2, and ADF-CN3, respectively, of *A. dahurica* var. *formosana*; lanes 8, 9, 10, and 11 correspond to samples AAN-PC, AAN-JN1, AAN-JN2, and AAN-GW, respectively, of *A. anomala*; and lanes 12, 13, 14, and 15 correspond to samples AJA-AW, AJA-CJ, AJA-HL, and AJA-PS, respectively, of *A. japonica*. Details of all of these 15 samples are listed in Table 1. Lanes M and N represent the 100 bp DNA ladder and no template control, respectively. Arrows indicate the sizes of the PCR products, and arrowheads indicate the sizes of different molecular weight bands of the DNA ladder.

To develop species-specific SCAR markers, comparative sequence analysis of the ITS regions of all plant samples was performed, and three species-specific primer pairs were designed (Table 3). The forward primers and reverse primers were designed using the ITS1 and ITS2 region, respectively. Hence, the resultant PCR amplicons contain the 5.8S ribosomal-RNA region (Figure 1). The size of the PCR fragments generated using these primers was unique for each species (Figure 2): 189, 259, and 309 bp for *A. dahurica* and *A. dahurica* var. *formosana*, *A. anomala*, and *A. japonica*, respectively (Figure 2).

### 2.3. Development of A Multiplex SCAR Assay for the Authentication of Herbal Medicines

**Figure 3:**
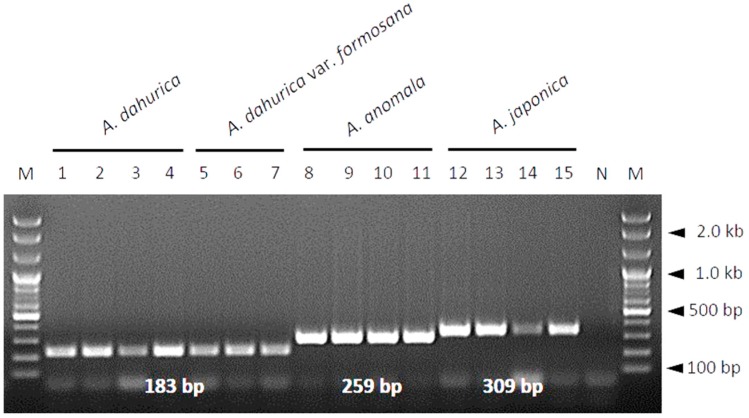
Gel image showing the multiplex SCAR assay developed in this study. PCR products were produced using three primer pairs (ADA-F/ADA-R, AAN-F/AAN-R, and AJA-F/AJA-R) in a single PCR reaction. Lanes 1–15 correspond to samples of *Angelica* species, as described in Figure 2. Lanes M and N represent the 100 bp DNA ladder and no template control, respectively. Sizes of PCR products are indicated on the image in white. Arrowheads indicate the sizes of different molecular weight bands of the DNA ladder.

**Figure 4:**
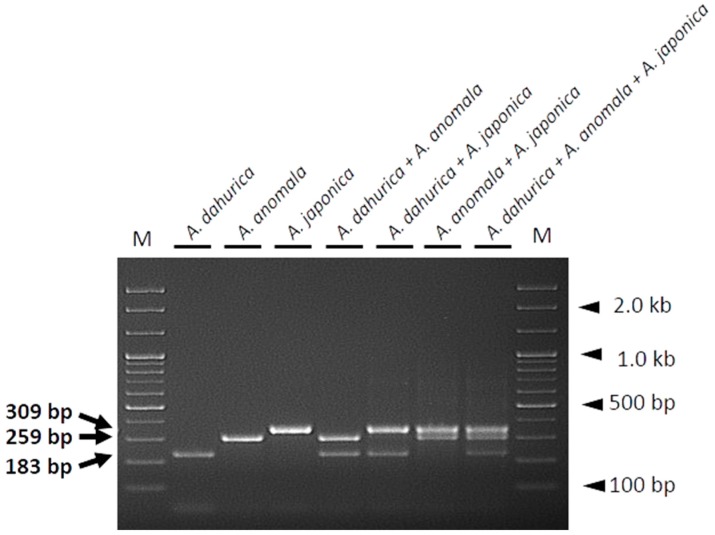
Gel image showing the effectiveness of the multiplex SCAR assay on DNAs of individual *Angelica* species (controls) and mixed template DNAs prepared by mixing the templates of two or more species. PCR products were produced using three primer pairs (ADA-F/ADA-R, AAN-F/AAN-R, and AJA-F/AJA-R) in a single PCR reaction. The template DNA used in each lane is indicated. Lane M represents a 100 bp DNA ladder. Arrows indicate the sizes of PCR products, and arrowheads indicate the sizes of different molecular weight bands of the DNA ladder.

Differences in PCR product sizes are key for species identification. In this study, we developed a multiplex SCAR assay to distinguish Angelicae Dahuricae Radix from other related species using all three species-specific SCAR primer pairs in a single PCR reaction. Each primer pair specifically amplified the DNA of the corresponding species, yielding distinct species-specific PCR products (Figure 3). Additionally, for the mixed template DNAs composed of two to three different species combinations (*A. dahurica* + *A. anomala*; *A. dahurica* + *A. japonica*; *A. anomala* + *A. japonica*; *A. dahurica* + *A. anomala* + *A. japonica*), authentic materials and adulterants were successfully detected at the species level, depending on the sizes of PCR products when PCR amplification was carried out using all three primer pairs (ADA-F/ADA-R, AAN-F/AAN-R, and AJA-F/AJA-R) in a single reaction. Thus, each PCR reaction containing mixed template DNAs produced distinct PCR products representative of the corresponding species (Figure 4). These data demonstrate that the primers designed in this study are capable of discriminating among the three *Angelica* species related to Angelicae Dahuricae Radix in a multiplex PCR.

**Figure 5:**
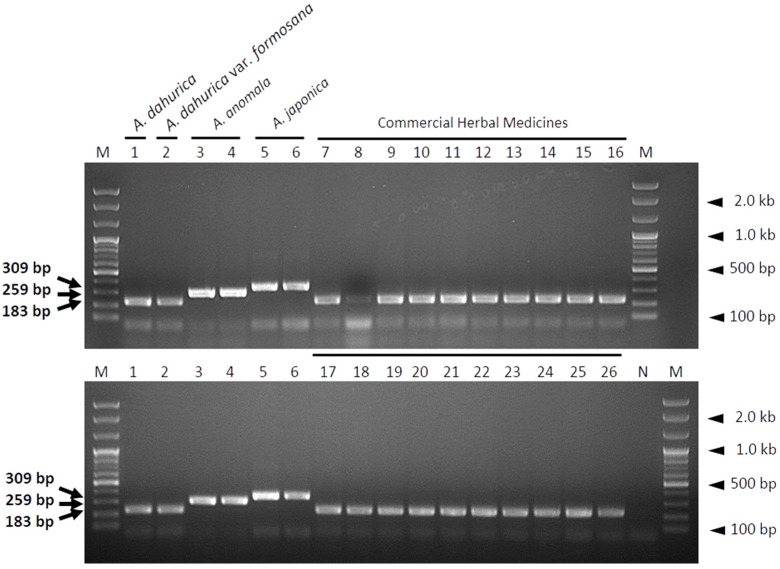
Identification of commercial Angelicae Dahuricae Radix samples using a multiplex SCAR assay. PCR products were produced using three primer pairs (ADA-F/ADA-R, AAN-F/AAN-R, and AJA-F/AJA-R) in a single PCR reaction. Lanes 1–6: Control plant samples; lanes 7–26: Commercial Angelicae Dahuricae Radix samples purchased from China and Korea (Table S1). Lanes M and N represent the 100 bp DNA ladder and no template control, respectively. Arrows indicate the sizes of PCR products, and arrowheads indicate the sizes of different molecular weight bands of the DNA ladder.

The multiplex SCAR assay was subsequently used to investigate the current commercial distribution status of Angelicae Dahuricae Radix, and to verify the reproducibility of the developed SCAR markers. A total of 20 commercial samples of Angelicae Dahuricae Radix were purchased from herbal markets in China and Korea (Table S1). Although the amplification signal intensity of one commercial sample (sample number 8; Figure 5) was weaker than that of the other samples, all of the 20 commercial samples were identified as genuine Angelicae Dahuricae Radix (dried roots of *A. dahurica*) (Figure 5 and Table S1). These results suggest that the multiplex SCAR assay developed in this study is capable of rapidly and effectively discriminating between authentic Angelicae Dahuricae Radix and its related herbal species.

## 3. Discussion

The dried roots of *A. dahurica* and its variety *formosana*, collectively referred to as Angelicae Dahuricae Radix, are commonly used as a traditional herbal medicine. Related species of *A. dahurica*, including *A. anomala* and *A. japonica*, are morphologically similar in appearance to *A. dahurica*. *A. anomala* is considered as an important traditional medicinal plant and is used to treat several conditions, including inflammation, pain, and poisoning [27]. *Angelica japonica* has additionally been regarded as a potential herbal medicine, due to its antitumor actions [28]. However, *A. anomala* and *A. japonica* are not authorized as authentic medicinal plants, nor are they registered in the Korean Pharmacopoeia or the Pharmacopoeia of the People’s Republic of China, as their safety and usage have not yet been established [14,15,16,17]. 

The introduction of a SCAR marker based on DNA barcoding has been a key technique for the precise identification of medicinal plants at the species level, and for the authentication of the botanical origins of herbal medicines [29,30]. In order to develop SCAR markers for distinguishing *A. dahurica* and *A. dahurica* var. *formosana* from *A. anomala* and *A. japonica*, we amplified and sequenced the following candidate DNA barcodes: The ITS region of nuclear rDNA and the *matK*, *rbcL*, and *psbA–trnH* regions of chloroplast DNA. Using this DNA barcode sequence analyses, we developed three SCAR markers that can identify two authentic medicinal plant taxa for Angelicae Dahuricae Radix (*A. dahurica* and its variety *A. dahurica* var. *formosana*) and two adulterant species, *A. anomala* and *A. japonica*, from the ITS region of nuclear rDNA (Figure 1 and Figure 2). However, we could not develop SCAR markers to differentiate between *A. dahurica* and its variety *formosana*, because the nucleotide sequences of all four DNA barcodes were identical in *A. dahurica* and *A. dahurica* var. *formosana* (Figure 1 and Figure 2). These results indicate that these DNA barcode regions are not capable of distinguishing between *A. dahurica* and *A. dahurica* var. *formosana*, and strongly support previously reported results which reported that DNA barcoding is sufficient as a molecular marker at inter-specific and intergeneric levels [10,31,32,33]. However, we did not consider distinguishing these two authentic plant taxa because the roots of the two plant taxa are official herbal materials listed in the National Pharmacopoeias of Korea and China [15]. Additional genomic fingerprinting analyses, such as RFLP, RAPD, inter simple sequence repeats (ISSRs), or the next-generation sequencing of whole genomes, may be attempted to acquire genetic information for identifying *A. dahurica* and *A. dahurica* var. *formosana*. 

Sequence analyses of *A. dahurica*, *A. anomala*, and *A. japonica* showed the largest number of species-specific nucleotide polymorphisms (44 of 690 bp) in the ITS region, followed by *matK* (5 of 1276 bp), *psbA–trnH* (3 of 332 bp), and *rbcL* (2 of 1503 bp) (data not shown), suggesting a greater applicability of the ITS region as a DNA barcode than the chloroplast regions for discriminating among the three *Angelica* species, which is consistent with the report of Yuan et al. [25]. The ITS region is a popular DNA barcode for species identification due to its high inter-specific distance. However, the intra-specific distance of the ITS region is also high in comparison with other DNA barcode regions [33]. To avoid intra-specific nucleotide variation from being counted as a species-specific nucleotide polymorphism, we collected plant samples from at least three different sites (Table 1). In addition, we also confirmed that the nuclear rDNA ITS sequences of *A. dahurica* were identical to those registered by Yuan et al. (GenBank accession nos. JX022904, JX022905, and JX022940) and did not show any nucleotide sequence variability in the species-specific primer regions (data not shown) [25]. These results suggest that the SCAR primers to identify *A. dahurica* are stable to amplify authentic Angelicae Dahuricae Radix.

Although using DNA barcodes for species identification is generally advantageous, it has some drawbacks [10,29,34]. The principal requirement for DNA barcoding is high quality DNA [29]. During the processing of herbal medicines, DNA is likely degraded and fragmented due to drying at high temperature and extreme pH [29]. Poor DNA quality obstructs the amplification of DNA fragments of sufficient size for species identification [10,25,33,34]. In this respect, developing species-specific SCAR markers targeting short DNA sequences, rather than reading the complete sequence of DNA barcode regions, provides a more efficient method for the identification of herbal medicines [35]. We designed primers to target short fragments of the ITS region (<400 bp), which enable markers to successfully amplify low-quality DNA [31]. The primer pairs designed in this study successfully amplified species-specific DNA fragments of different sizes in both SCAR (Figure 2) and multiplex SCAR (Figure 3) assays. We verified the ability of the multiplex SCAR assay to amplify and discriminate among a mixture of template DNAs (Figure 4), and used the assay to analyze commercially distributed Angelicae Dahuricae Radix (Figure 5). From the verification of the botanical origins of 20 samples of commercially processed Angelicae Dahuricae Radix purchased from Korean and Chinese herbal markets, we did not confirm adulteration or contamination (Table S1 and Figure 5). To verify the discriminability of SCAR markers, PCR products amplified from commercial samples of Angelicae Dahuricae Radix using the multiplex SCAR assay were sequenced to confirm the botanical origins and sequence identities of the samples. The sequences of all the commercial samples were identical to those of the control plants (data not shown). Although we did not confirm the adulteration or contamination of inauthentic herbal materials originating from *A. anomala* and *A. japonica* in this study, additional consistent monitoring assays are needed to prevent the adulteration and contamination of Angelicae Dahuricae Radix in herbal markets. The commercial sample number 8 obtained from the Chinese herbal medicine market showed weak amplification intensity in the multiplex SCAR assay (Figure 5). It is possible that this sample was subjected to harsh treatment, such as hot air drying during processing, which degraded its DNA. Overall, we predict that the multiplex SCAR assay developed in this study will prove to be advantageous in reducing both the time and cost involved in DNA barcoding, thus allowing researchers to discriminate between genuine Angelicae Dahuricae Radix and adulterants.

## 4. Materials and Methods 

### 4.1. Plant Material and Herbal Medicines

Three or four plant samples each of *A. dahurica*, *A. dahurica* var. *formosana*, *A. anomala*, and *A. japonica* were used in this study (Table 1). The scientific name of the plant was listed in accordance with The Plant List (http://www.theplantlist.org/). All plant samples were collected from multiple native habitats or farming fields in Korea and China, and stored at a temperature of −70 °C before further analysis. Commercially available samples of herbal medicine were purchased from various herbal markets across Korea and China. Plant samples of *A. dahurica*, *A. anomala*, and *A. japonica* were saved as specimens, and deposited in the Korean Herbarium of Standard Herbal Resources (Index Herbariorum code KIOM) under unique voucher numbers (Table 1).

### 4.2. Genomic DNA Extraction

Genomic DNA was extracted from frozen leaves or dried specimens using a DNeasy^®^ Plant Mini Kit (QIAGEN, Valencia, CA, USA), according to the manufacturer’s instructions, and stored at a temperature of −20 °C. The concentration of the DNA samples was measured using a spectrophotometer (Nanodrop ND-1000, Nanodrop, Wilmington, DE, USA), and the final concentration of all DNA samples was adjusted to approximately 15 ng/μL for PCR amplification.

### 4.3. PCR Amplification and Sequencing of The ITS Region

The ITS region was amplified using the universal primers ITS1 (5′-TCCGTAGGTGAACCTGCGG-3′) and ITS4 (5′-TCCTCGCTGATTGATATGC-3′) [36]. PCRs were performed in a total reaction volume of 40 μL, comprising 10mM Tris-HCl (pH 9.0), 2.5 mM MgCl_2_, 200 μM of each dNTP, 10 mM (NH_4_)_2_SO_4_, 0.5 U Taq DNA polymerase (Solgent, Daejeon, Korea), 0.5 μM of each primer, and 15 ng of template DNA, on a ProFlex PCR System (Applied Biosystems, Life Technologies, Foster City, CA, USA). The following conditions were used for PCR amplification: An initial denaturation at a temperature of 95 °C for 2 min, followed by 35 cycles of denaturation at a temperature of 95 °C for 30 s, annealing at a temperature of 55 °C for 30 s, an extension at a temperature of 72 °C for 45 s, and a final extension at a temperature of 72 °C for 5 min. The PCR products were separated by gel electrophoresis on a 1.5% agarose gel alongside a 100 bp DNA ladder (Solgent), and visualized using Ecodye™ Nucleic Acid Staining Solution (Biofact, Daejeon, Korea). The identifiable PCR products were extracted from the agarose gel using a QIAquick gel extraction kit (QIAGEN, Valencia, CA, USA). The nuclear rDNA-ITS regions extracted from the agarose gel were sub-cloned into the pGEM-T Easy Vector (Promega, Madison, WI, USA) and transformed into *Escherichia coli* JM109 competent cells (RBC, Taipei, Taiwan), following the manufacturer’s instructions [37]. The transformed cells were plated on Luria Broth (LB) agar media containing 100 μg/mL ampicillin, 40 μg/mL X-gal, and 0.5 mM isopropyl β-d-1-thiogalactopyranoside (IPTG), and incubated at a temperature of 37 °C for 18 h. Recombinant clones were confirmed by colony PCR using vector-based primers, T7 and SP6. At least three white colonies for each sample were sequenced using the Sanger method.

### 4.4. Nucleotide Sequence, Phylogenetic Analyses and SCAR Marker Development

To avoid errors during PCR amplification and sequencing, and to identify potential chimeric sequences, the sequence quality was checked by comparing sequences from three white colonies from each sample. The ITS sequences were verified using the NCBI BLAST search tool, and deposited in the GenBank database under the following accession numbers: *A. dahurica*, MH188430–MH188433; *A. dahurica* var. *formosana*, MH188434–MH188436; *A. anomala*, MH188437–MH188440; and *A. japonica*, MH188441–MH188444. 

Approximately 700 bp of all 15 ITS sequences were aligned using ClustalW, and manually edited using BioEdit ver. 7.2.5 (North Carolina State University, Raleigh, NC, USA) [38] to identify species-specific indels and nucleotide substitutions. Inter- and intra-species genetic distances were calculated using MEGA ver. 7.0, following the K2P model with 1000 bootstrap replicates [39]. A best-fit substitution model was selected using MEGA for phylogenetic analysis, and a phylogenetic tree was constructed using the ML method using MEGA, based on the selected model, using all gaps or missing data. The ITS sequence of *Heracleum moellendorffii* Hance (GenBank accession number: MH188445) served as an outgroup in the phylogenetic analysis. 

To develop SCAR markers, species-specific primer pairs were designed using species-specific nucleotide substitutions to amplify short DNA fragments of different sizes for each species (Table 3). PCRs were conducted in a total reaction volume of 30 μL, comprising 10mM Tris-HCl (pH 9.0), 2.5 mM MgCl_2_, 200 μM of each dNTP, 10 mM (NH_4_)_2_SO_4_, 0.5 U Taq DNA polymerase (Solgent), 0.5 μM of each species-specific forward and reverse primers (Table 3), and 15 ng of template DNA, using the following conditions: An initial denaturation at a temperature of 95 °C for 2 min, followed by 35 cycles of denaturation at a temperature of 95 °C for 30 s, annealing at a temperature of 68 °C for 30 s, an extension at a temperature of 72 °C for 40 s, and a final extension at a temperature of 72 °C for 5 min. The PCR products were visualized by gel electrophoresis on a 1.5% agarose gel, as described in Section 4.3.

### 4.5. Development of Multiplex SCAR Assay and Monitoring of Commercial Herbal Medicines

To develop the multiplex SCAR assay, all three species-specific primer pairs were combined in a single PCR reaction. Optimal PCR conditions were determined by altering various parameters, including the duration and temperature of annealing, the number of amplification cycles, the concentrations of primer, and the combinations and concentrations of template DNAs. The quality of PCR products was verified by gel electrophoresis on a 1.5% agarose gel, as described in Section 4.3.

To validate the multiplex PCR method and to verify the authenticity of the commercial Angelicae Dahuricae Radix samples, 20 commercially available Angelicae Dahuricae Radix samples were purchased and examined using the multiplex SCAR assay. Approximately 15 ng of genomic DNA was used in a 30 μL PCR reaction, comprising 10 mM Tris-HCl (pH 9.0), 2.5 mM MgCl_2_, 200 μM of each dNTP, 10 mM (NH_4_)_2_SO_4_, 0.5 U Taq DNA polymerase (Solgent), 0.5 μM of each three species-specific primer pair, and 15 ng of template DNA. The protocol used for amplification in the multiplex PCR was the same as that described in Section 4.4. The quality of PCR products was verified by gel electrophoresis on a 1.5% agarose gel, as described in Section 4.3.

To examine the ability of SCAR markers to identify adulterants in processed herbal medicines, template DNAs of two or more *Angelica* species were mixed in a 1:1 ratio (*w*:*w*) and subjected to multiplex PCR, as described in Section 4.5. The PCR products were separated by gel electrophoresis on a 2% agarose gel and visualized using Ecodye™ Nucleic Acid Staining Solution (Biofact).

## Figures and Tables

**Table 1:** Details of the *Angelica* species investigated in this study.

Species	Herbal Name	Collection Site	Collection Date	Voucher Number	Abbreviation
*A. dahurica* (Hoffm.) Benth. & Hook.f. ex Franch. & Sav.	Angelicae Dahuricae Radix	Beonam, Jangsu, Jeonbuk, Korea	2015-09-11	KIOM201501015740	ADA-JS
		Iwon, Taean, Chungnam, Korea	2015-08-25	KIOM201501015772	ADA-TA
		Punggi, Yeongju, Gyeongbuk, Korea	2017-08-09	KIOM201801020615	ADA-YJ
		Gohan, Jeongseon, Gangwon, Korea	2017-08-10	KIOM201801020618	ADA-JN
*A. dahurica* var. *formosana* (Boissieu) Yen	Angelicae Dahuricae Radix	Nangang, Harbin, Heilongjiang, China	2014-08-06	2014CHINA1-1	ADF-CN1
			2014-08-06	2014CHINA1-2	ADF-CN2
			2014-08-06	2014CHINA1-3	ADF-CN3
*A. anomala* Avé-Lall.	-	Bongpyeong, Pyeongchang, Gangwon, Korea	2015-07-29	KIOM201501015646	AAN-PC
		Gohan, Jeongseon, Gangwon, Korea	2017-08-10	KIOM201801020679	AAN-JN1
		Gohan, Jeongseon, Gangwon, Korea	2017-08-10	KIOM201801020685	AAN-JN2
		Gunwi, Gyeongbuk, Korea	2013-07-14	KIOM201501011489	AAN-GW
*A. japonica* A. Gray	-	Aewol, Jeju, Jeju, Korea	2007-03-30	KIOM201501011678	AJA-AW
		Chuja, Jeju, Jeju, Korea	2015-07-15	KIOM201501015106	AJA-CJ
		Hallim, Jeju, Jeju, Korea	2016-12-05	KIOM201701019409	AJA-HL
		Pyoseon, Seogwipo, Jeju, Korea	2016-12-06	KIOM201801020395	AJA-PS

**Table 2:** Characteristics of internal transcribed spacer (ITS) barcode sequences.

Species	Length of ITS (bp)	Aligned Length (bp)	Intra-Species Variability (%) ^1^	Inter-Species Variability (%) ^1^	Species-Specific Mutations	
					Indels	Substitutions
*A. dahurica*	689	690	0.0000 ± 0.0000	0.0476 ± 0.0053	0	18
*A. dahurica* var. *formosana*						
*A. anomala*	689	690	0.0017 ± 0.0012	0.0423 ± 0.0021	0	10
*A. japonica*	690	690	0.0015 ± 0.0010	0.0461 ± 0.0074	1	16

^1^ Data represent mean ± standard deviation (SD).

**Table 3:** List of primers used in this study for the development of SCAR markers.

Species	Primer Name ^1^	Primer Sequence (5′→3′)	PCR Product Size (bp)
*A. dahurica*	ADA-F	ATCGGCGTCTTTCCAAAATGC	183
*A. dahurica* var. *formosana*	ADA-R	GCACAACTTCTCAGGTGTGCCT	
*A. anomala*	AAN-F	AAAATCATTCAGGCGCGGAGAG	259
	AAN-R	AAACCGGCACAACTTCTCATGT	
*A. japonica*	AJA-F	GGCCACTCCTGGGTGGCCAGAG	309
	AJA-R	CGGGAGGCCAGTTTCCGCCAGA	

^1^ F and R in primer names indicate the forward and reverse primers, respectively.

## Supplementary Materials

The following are available online, Figure S1: A phylogenetic tree showing the relationships among 15 samples of three *Angelica* species based on sequences of the internal transcribed spacer (ITS) regions; Table S1: A list of plant samples and commercial herbal medicines investigated using a multiplex SCAR assay developed in this study.
