# Supplementary material for: Authentication of the Herbal Medicine Angelicae Dahuricae Radix Using an ITS Sequence-Based Multiplex SCAR Assay
Source: Molecules. 2018 Aug 24;23(9):2134. doi: 10.3390/molecules23092134 (PMC6225120; doi:10.3390/molecules23092134)
Supplement: Supplementary file 1 [file molecules-23-02134-s001.pdf]

Supplementary Materials: Authentication of the Herbal Medicine Angelicae Dahuricae Radix Using an ITS Sequence-Based Multiplex SCAR Assay

Pureum Noh, Wook Jin Kim, Sungyu Yang, In Kyu Park and Byeong Cheol Moon

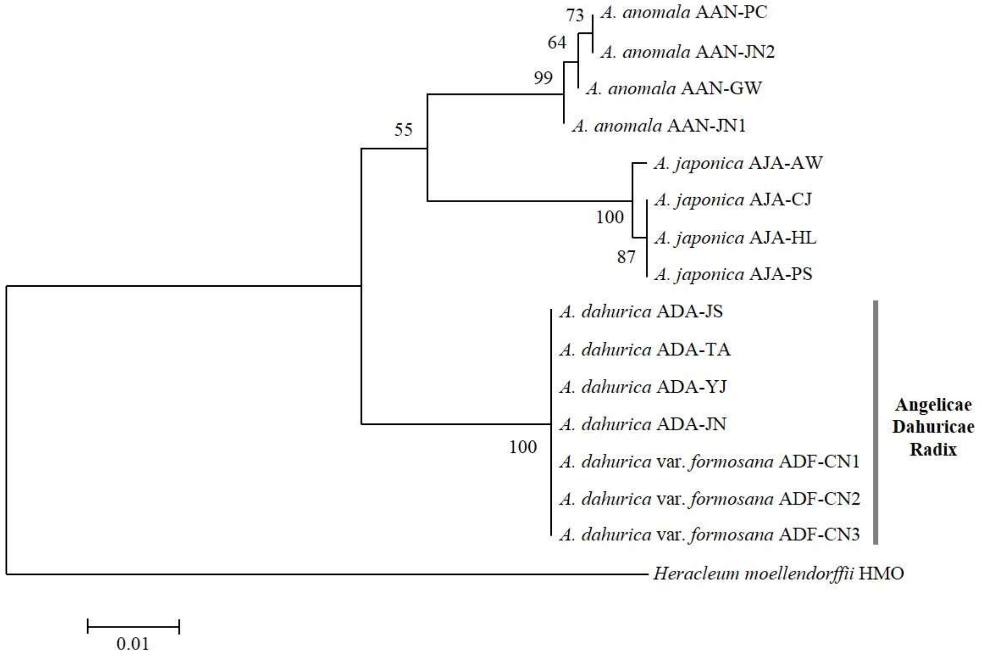

Figure S1. Phylogenetic tree showing the relationship among 15 samples of three *Angelica* species based on sequences of the internal transcribed spacer (ITS) regions. The tree was constructed using the maximum likelihood method, with 1000 bootstrap replicates. Bootstrap values are indicated at the nodes. The ITS sequence of *Heracleum moellendorffii* Hance (GenBank accession number: MH188445) served as an out-group.

Table S1. List of plant samples and commercial herbal medicines investigated using a multiplex SCAR assay developed in this study.

Sample Name	Origin	Manufacturer	Collection Date	Lane in Gel (Figure 5)
A. dahurica	Beonam, Jangsu, Jeonbuk, Korea	-	2015-09-11	1
A. dahurica var. *formosana*	Nangang, Harbin, Heilongjiang, China	-	2014-08-06	2
A. anomala	Bongpyeong, Pyeongchang, Gangwon, Korea	-	2015-07-29	3
	Gohan, Jeongseon, Gangwon, Korea	-	2017-08-10	4
A. japonica	Chuja, Jeju, Jeju, Korea	-	2015-07-15	5
	Hallim, Jeju, Jeju, Korea	-	2016-12-05	6
Angelicae Dahuricae Radix	Unidentified	Unidentified	2017-06-20	7
	Sichuan, China	S○○ pharmaceutical company	2015-01-08	8
	Gunwui, Gyeongbuk, South Korea	D○○ pharmaceutical company	2014-07-07	9
	China	D○○ pharmaceutical company	2014-07-07	10
	China	G○○ pharmaceutical company	2014-07-07	11
	South Korea	G○○ pharmaceutical company	2014-07-07	12
	Gyeongbuk, South Korea	D○○ pharmaceutical company	2009-09-07	13
	Jungsun, Gangwon, South Korea	U○○ pharmaceutical company	2017-10-11	14
	Yeongyang, Gyeongbuk, South Korea	G○○ pharmaceutical company	2017-10-11	15
	Zhejiang, China	G○○ pharmaceutical company	2017-10-11	16
	Gyeongbuk, South Korea	D○○ pharmaceutical company	2017-10-11	17
	China	D○○ pharmaceutical company	2017-10-11	18
	China	Yak Jae ○○	2017-10-11	19
	Jeonnam, South Korea	Ji Ri San ○○	2017-10-11	20
	Bonghwa, Gyeongbuk, South Korea	Bon Cho ○○	2017-10-11	21
	South Korea	Dong Gwang ○○	2017-10-11	22
	Yeongju, Gyeongbuk, South Korea	Hyo Sa Mo ○○	2017-10-11	23
	South Korea	Jung Woo ○○	2017-10-11	24
	Bonghwa, Gyeongbuk, South Korea	Seo Hyeon ○○	2017-10-11	25
	Hoengseong, Gangwon, South Korea	Yong Sam ○○	2017-10-11	26
